# Multi-genome identification and characterization of chlamydiae-specific type III secretion substrates: the Inc proteins

**DOI:** 10.1186/1471-2164-12-109

**Published:** 2011-02-16

**Authors:** Pierre Dehoux, Rhonda Flores, Catherine Dauga, Guangming Zhong, Agathe Subtil

**Affiliations:** 1 Institut Pasteur, Génopôle, Plate-forme Intégration et Analyse génomique, Paris, France; 2 Department of Microbiology and Immunology, University of Texas Health Science Center at San Antonio, 7703 Floyd Curl Drive, San Antonio, TX78229, USA; 3 Institut Pasteur, Unité de Biologie des Interactions Cellulaires, Paris, France; 4 CNRS URA 2582, Paris, France

## Abstract

**Background:**

*Chlamydiae *are obligate intracellular bacteria that multiply in a vacuolar compartment, the inclusion. Several chlamydial proteins containing a bilobal hydrophobic domain are translocated by a type III secretion (TTS) mechanism into the inclusion membrane. They form the family of Inc proteins, which is specific to this phylum. Based on their localization, Inc proteins likely play important roles in the interactions between the microbe and the host. In this paper we sought to identify and analyze, using bioinformatics tools, all putative Inc proteins in published chlamydial genomes, including an environmental species.

**Results:**

Inc proteins contain at least one bilobal hydrophobic domain made of two transmembrane helices separated by a loop of less than 30 amino acids. Using bioinformatics tools we identified 537 putative Inc proteins across seven chlamydial proteomes. The amino-terminal segment of the putative Inc proteins was recognized as a functional TTS signal in 90% of the *C. trachomatis *and *C. pneumoniae *sequences tested, validating the data obtained *in silico*. We identified a *macro *domain in several putative Inc proteins, and observed that Inc proteins are enriched in segments predicted to form coiled coils. A surprisingly large proportion of the putative Inc proteins are not constitutively translocated to the inclusion membrane in culture conditions.

**Conclusions:**

The Inc proteins represent 7 to 10% of each proteome and show a great degree of sequence diversity between species. The abundance of segments with a high probability for coiled coil conformation in Inc proteins support the hypothesis that they interact with host proteins. While the large majority of Inc proteins possess a functional TTS signal, less than half may be constitutively translocated to the inclusion surface in some species. This suggests the novel finding that translocation of Inc proteins may be regulated by as-yet undetermined mechanisms.

## Background

Members of the phylum *Chlamydiae *form a phylogenetically well-isolated group of bacteria. It includes the family *Chlamydiaceae*, which are pathogenic bacteria infecting a wide range of Vertebrates, as well as symbionts of free-living amoebae and other eukaryotic hosts, often referred to as environmental chlamydiae [1]. The most prominent member of the phylum is *Chlamydia trachomatis*, an exclusively human pathogen, which is the leading cause of preventable blindness and of sexually transmitted diseases of bacterial origin [2,3]. The other important species for public health is *Chlamydia pneumoniae*, a causative agent of pneumoniae, which has also been associated with a number of chronic diseases such as atherosclerosis, adult-onset asthma and Alzheimer's disease [4]. Although not clearly documented, a role for environmental chlamydiae in human diseases cannot be excluded.

In addition to relatedness at the genomic level, members of the phylum share two characteristics: an obligate intracellular lifestyle and a unique biphasic developmental cycle [5]. Infection starts with the attachment of the infectious form of the microorganism, the elementary body, to a eukaryotic host cell. Upon attachment, intracellular signaling events lead to the internalization of the bacterium in a membrane-bound compartment called an inclusion. Importantly, the remainder of the developmental cycle takes place inside this compartment. Internalized, infectious particles differentiate immediately to metabolically active bacteria, or reticulate bodies, which replicate in the inclusion. At the end of the developmental cycle, the bacteria differentiate back into elementary bodies that are released to the extracellular space to initiate a new infectious cycle.

The inclusion membrane is a key player in the interactions between chlamydiae and the host cell. Its composition dictates the exchanges between the lumen of the inclusion, in which the bacteria reside, and the host cytoplasm. Microscopy studies indicate that chlamydiae incorporate membranes from several intracellular compartments [6-9]. However, very few eukaryotic proteins have been shown to be in the inclusion membrane. In contrast, many different proteins of bacterial origin have been found in this location. The first one, IncA, was isolated based on its immunogenicity, as antibodies against this protein were abundant in sera of convalescent guinea pigs [10]. Subsequently, homologs of IncA have been found in all *Chlamydiaceae *species, and the protein was shown to play a central role in controlling the fusion of inclusions and the interactions between the inclusion and intracellular compartments [11-13]. Following the discovery of IncA, other inclusion membrane proteins were identified and designated as Inc proteins (Inclusion proteins) [14,15]. In addition to their localization to the inclusion membrane, they share a feature that became a hallmark of the family: a large hydrophobic domain of 40 to 60 residues with hydrophilic residues in its middle, giving it a bilobal pattern on hydropathy plots. Access to genome sequences of chlamydiae revealed an abundance of proteins with such a profile. A manual approach identified 46 *C. trachomatis *and 70 *C. pneumoniae *proteins with one or two bilobal hydrophobic domain [16]. Antibodies against five out of six predicted members of the Inc family demonstrated their localization to the inclusion membrane, thus confirming their designation as Inc family members. Three years later, based on the 13 Inc proteins identified at the time, a second study used an *in silico *approach to predict Inc proteins in the same two human pathogens. Based on somewhat different criteria, this second list differs slightly from the first one, but confirms the specificity of Inc proteins to *Chlamydiae *genomes and the extension of the family in *C. pneumoniae *compared to *C. trachomatis *[17]. To date, *C. trachomatis *is by far the species for which the Inc catalog is best characterized, with about twenty members [18]. Only a handful has been characterized using specific antibodies in other species, including very recently the environmental species *Protochlamydia amoebophila *(see references in Table 1).

**Table 1:** Chlamydial Inc proteins observed on the inclusion membrane using specific antibodies

Name	Ortholog Group # (this study)	Proposed Partner/Function	Reference
CAB0766	842		[73]

CCA0491 (IncB)	79		[14]

CF0218	842		[74]

CPn0146	287		[37]

CPn0147	455		[37]

CPn0186	264		[16]

CPn0308	No ortholog		[75]

CPn0517 (CP0236)	No ortholog	Act1 [22]	[22]

CPn0585	842	Rab GTPases[20]	[76]

CPn1027	No ortholog		[77]

CT101	236		[35]

CT115 (IncD)	987		[15]

CT116 (IncE)	982		[15]

CT117 (IncF)	961		[15]

CT118 (IncG)	988	14-3-3 β [21]	[15]

CT119 and CCA0550 (IncA)	264	IncA and SNARE domains [13,78]	[10,11]

CT147	553	Similarity to EEA1[26]	[26]

CT222	No ortholog		[35]

CT223	842		[16]

CT225	No ortholog		[18]

CT226	979		[66]

CT228	964		[18]

CT229	965	Rab4 [79]	[16]

CT233 and CCA0490 (IncC)	26		[14,23]

CT249	966		[80]

CT288	774		[16]

CT358	968		[18]

CT440	411		[18]

CT442	515		[16]

CT618	612		[81]

CT813	978	SNARE domains [13]	[65]

CT850	151		[35]

pc0156	344		[82]

pc0399	No ortholog		[82]

pc0530	No ortholog		[82]

pc1111	No ortholog		[82]

The bilobal hydrophobic domain of Inc proteins is predicted to enable its insertion into the inclusion membrane, although, in the absence of genetic tools to manipulate chlamydiae, it is difficult to demonstrate. Furthermore, it is assumed that at least one segment of the protein faces the cytosol of the host. This has been demonstrated in a few cases, either directly by microinjecting antibodies into the cytoplasm, or indirectly by identifying eukaryotic partners [19-22]. Type III secretion (TTS) signals have been found in the amino terminal domain of several Inc proteins, indicating that this is the secretion mechanism used to transit the bacterial outer membranes [23,24]. The precise mechanism by which the proteins, following transit through the secretion needle, are inserted in the inclusion membrane is unknown.

From their localization at the interface between the bacteria and the host, Inc proteins are expected to be involved in varied processes. However, only a few interactions between Inc proteins and eukaryotic proteins have been described (listed with references in Table 1), and, for the most part, their exact function in infection is totally unknown. Genes coding for the Inc proteins are not all expressed at the same time during the developmental cycle [25,26], indicating that the proteins participate in the maturation of the inclusion membrane and might be only transiently present on the membrane, fulfilling a function limited to certain stages of development. Early comparisons of the putative Inc proteins of *C. trachomatis *and *C. pneumoniae *indicated that only a subset is conserved between these two species [16]. For those which are conserved, the level of similarity is usually very low, and the Inc proteins are among the least conserved proteins when comparing the two species. This is somewhat surprising if Inc proteins are involved in key interactions between the bacteria and the host, which are expected to be conserved in all *Chlamydiaceae *species. One partial answer to that intriguing question may come from the observation that many of the Inc proteins are immunogenic during *C. trachomatis *infection in humans [18]. To counterbalance their exposure to the host immune system, genes coding for Inc proteins might be subjected to a higher rate of modification than the rest of the genome.

Since the early manual description of putative Inc proteins in the *C. trachomatis *and *C. pneumoniae *proteomes, seven different species of *Chlamydiaceae *have been sequenced, including one species of an environmental chlamydiae, *Protochlamydia amoebophila*. Furthermore, more than thirty putative Inc proteins have now been validated using specific antibodies, mostly in *C. trachomatis *(Table 1). We used this information to identify features characteristic of all Inc proteins, which we used in a systematic computer-based approach to identify all putative Inc proteins in published chlamydial genomes. Using a heterologous secretion assay in *Shigella*, we observed that a large majority of these proteins contained a functional TTS signal. This result validated our criteria for the *in silico *identification of Inc proteins. In spite of their ability to be recognized as TTS substrates, many putative Inc proteins are not detected at the inclusion membrane during *in vitro *culture, suggesting that their translocation might be controlled by an unknown mechanism.

## Results

### Inc protein hydrophobic domains consist of two transmembrane alpha helices

**Figure 1:**
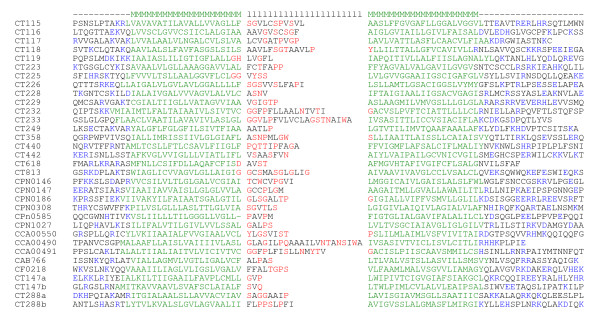
**Composition of the bilobal hydrophobic domain of known Inc proteins**. Bilobal domains were aligned manualy utilizing the topological information obtained from Topcons. Identifiers with a and b correspond to the first bilobal (a) and second bilobal (b) domains of CT147 and CT288. Transmembrane residues are in green, flanking regions charged residues in blue, residues with a high potential to form turns in red (turn propensity scale: P>N>R>D>Q>H>K>E>G>W>S>Y>T|C M I V A F L[30]). M = transmembrane domain, l = loop domain. Note that transmembre domain limits were predicted by Topcons and may vary from the limits predicted by Polyphobius and reported in Tables 2, 3 and in the Additional files 1, 2, 3, 4, 5.

The hallmark of Inc proteins is a large hydrophobic domain of 40 to 60 residues with non-hydrophobic residues in its middle, resulting in a bilobal pattern on hydropathy plots [16]. While it is assumed that this hydrophobic domain serves as an anchor in the inclusion membrane, its secondary structure has not been investigated. The most common secondary structure for transmembrane segments is the alpha helix; other structures include short buried hydrophobic helices or beta-barrels [27]. We submitted the sequences of 31 known Inc proteins (listed in Figure 1) to Split analysis, which predicts the secondary structure of the transmembrane domains of membrane proteins [28]. In all cases, Split analysis predicted that Inc protein hydrophobic domains correspond to two alpha helical transmembrane segments, ranging from 15 to 32 residues, connected with a short loop of 3 to 22 residues.

The proximity of two helical segments suggested that they might constitute transmembrane helical hairpins, which consist of two closely spaced transmembrane helices separated by a tight turn loop with charged residues in the flanking regions [29,30]. To test this hypothesis, the sequences of known Inc proteins were submitted to Topcons, which established a consensus prediction of membrane protein topology based on different programs and allowed us to define the limits of the helices and of the loop [31]. Amino acids found in the loop between the helices were then subjected to the "turn propensity scale" of helical hairpins [30]. Residues known as turn-forming residues were enriched in the loop. Interestingly, helix-breaking Pro and Gly residues were over-represented as were the polar amino acid Asn and semi-polar Ser and Thr residues, whereas high turn-forming charged residues Lys, Arg, Asp, Glu were absent (Figure 1).

In conclusion, the length and composition of known Inc proteins are compatible with the topology observed for transmembrane domains separated by a loop [32]. Loop length was on average of 8 residues, however a minority of Inc proteins such as IncB and IncC presented a notably longer loop (15-22 residues). Most Inc proteins identified so far have only one transmembrane helical hairpin, with the exception of CT147, CT288 and CT850, which have two.

### *In silico *identification of all putative *inc *genes in seven chlamydial genomes

**Figure 2:**
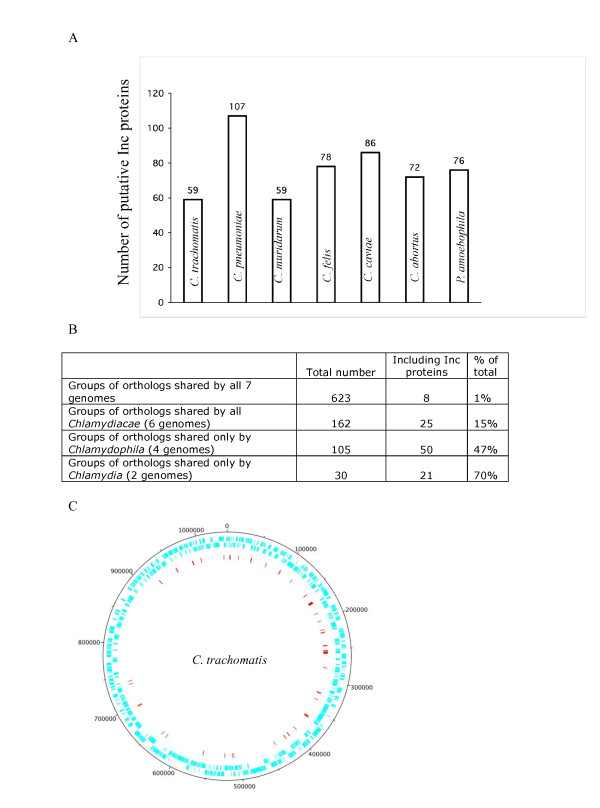
**Distribution of the putative Inc proteins among the seven genomes**. A. Numbers of putative Inc proteins in each genome. B. Conservation of putative Inc proteins between genomes based on groups of orthologs shared by all *Chlamydiales*, *Chlamydiaceae *only (not found in *P. amoebophila*), *Chlamydophila *only (not found in *P. amoebophila *or *Chlamydia)*, *Chlamydia *only (not found in *P. amoebophila *or *Chlamydophila)*. C. Distribution of putative *inc *genes on *C. trachomatis *genome. The genes are represented in blue on two circles, representing the two coding strands. Red lines indicate the position of the putative *inc *genes. The figure was constructed using DNA Plotter [72].

To systematically identify all *inc *genes in fully sequenced *Chlamydiae *genomes we designed a biocomputational approach based on the presence, in all Inc proteins, of at least two transmembrane domains separated by a loop region ([16-18] and this study). Because the maximum size of the loop region of known Inc proteins is 22 amino acids (Figure 1), we set a threshold of 30 residues between the two transmembrane segments. *Chlamydiae *membrane proteins were collected from all 7 chlamydial proteomes using the Polyphobius predictor algorithm [33]. Out of the 2904 sequences obtained, we eliminated the sequences that only contained one hydrophobic N-terminus fragment identified as a signal peptide or that contained a single transmembrane domain. The remaining polytopic membrane proteins (1387 sequences) were submitted to the domain recognition program rpsblast associated with the NCBI-CDD database. Proteins generating multi-domain family hits (COG, TIGRfam), highly indicative of a conserved prokaryotic function, were removed. In addition, sequences containing a single domain covering the whole length of the protein were analyzed with Blast. Among those, we retained as candidates only the proteins specific to the chlamydial genus. Finally at this stage, only sequences containing at least one set of transmembrane domains separated by a loop of less than 30 amino acids were retained. Altogether, the seven chlamydial proteomes generated 537 sequences that fulfilled these criteria. The number of putative Inc proteins per chlamydial species ranges from 76 out of 2031 proteins (4%) in *P. amoebophila *to 107 out of 1052 proteins (10%) in *C. pneumoniae *(Figure 2A). The list of putative *C. trachomatis *and *C. pneumoniae *Inc proteins are shown in Table 2 and 3, respectively, while putative Inc proteins from other genomes are found in Additional files 1, 2, 3, 4 and 5.

**Table 2:** *C. trachomatis *D/UW-3/CX putative Inc proteins.

Sequence ID	0rtholog Group # (1)	Length (aa)	TM segments (2)	TM coordinates (3)	Additional feature(s)
CT005	667	363	4	[34-54]**6[**60-81]**5**[95-119]**6[**124-146]	

CT006	485	189	3	[89-112]**5**[117-138]	

CT018	714	157	3	[91-105]**2**[107-121]	

CT036	989	403	2	[28-50]**6**[56-75]	

CT058	722	367	2	[26-48]**5**[53-76]	Macro domain

CT079	580	147	2&SP	[SP 1-13][35-68]**13**[81-102]	

CT081	983	98	2	[39-58]**12**[70-96]	

CT101	236	153	3&SP	[SP 1-25]-[38-56] & [95-113]**6**[119-138]	

CT115	987	141	2	[37-61]**7**[68-93]	Inclusion Membrane Protein D (IncD)

CT116	982	132	2	[36-59]**5**[64-87]	Inclusion Membrane Protein E (IncE)

CT117	961	104	2	[38-62]**8**[70-91]	Inclusion Membrane Protein F (IncF)

CT118	988	167	2	[33-57]**6**[63-88]	Inclusion Membrane Protein G (IncG)

CT119	264	273	2	[35-59]**5**[64-84]	Inclusion Membrane Protein A (IncA) extended coiled coils

CT134	980	137	3	[80-98]**7**[105-122]	

CT135	713	360	3	[210-236]**6**[242-268]	

CT147	553	1449	4	[79-99]**6**[105-124] & [849-870]**6**[876-896]	extended coiled coils

CT164	No ortholog	86	2	[31-53]**7**[60-80]	

CT179	682	170	2	[2-19]**11[**30-52]	

CT192	969	257	2	[58-81]**6**[87-108]	

CT195	132	363	6	[53-74]**7**[81-104] & [173-194]**2**[196-215] & [238-252]**2**[254-268]	

CT196	977	106	2	[34-57]**6**[63-85]	

CT214	877	547	3	[36-58]**6**[64-89]	

CT222	No ortholog	129	2	[39-63]**6**[69-93]	

CT223	842	270	3	[38-61]**6[**67-91] & [180-194]	extended coiled coils

CT224	No ortholog	147	2	[35-58]**8**[66-87]	

CT225	No ortholog	122	2	[12-38]**6**[44-66]	

CT226	979	176	2	[45-68]**6**[74-99]	coiled coils and Leucine zipper

CT227	963	133	2	[36-64]**2**[66-88]	

CT228	964	196	2	[38-59]**6**[65-86]	coiled coils

CT229	965	215	2	[42-65]**6**[71-90]	coiled coils

CT232	79	115	2	[31-61]**6**[67-90]	

CT233	26	178	2	[100-127]**13**[140-164]	Inclusion Membrane Protein B (IncB)

CT249	966	116	2	[51-72]**6**[78-97]	Inclusion Membrane Protein C (IncC)

CT288	774	563	4	[36-58]**7[**65-88] & [242-263]**6**[269-291]	coiled coils

CT300	990	115	2	[34-54]**12**[66-95]	

CT324	772	303	3	[75-93]**6**[99-119]	

CT326	462	563	4	[323-337]**2**[339-353]**12**[365-382] & [438-461]	DUF687

CT345	972	121	2	[31-53]**6**[59-82]	

CT357	986	110	2	[37-58]**6**[64-86]	

CT358	968	178	2	[45-70]**6**[76-100]	

CT365	141	575	10	[54-77]**6**[83-105]**20**[125-146]**15[**161-179]**29 **[208-233]**6**[239-262] & [338-360]**6**[366-392] & [471-491]**6**[497-517]	

CT383	238	243	2	[104-130]**6[**136-157]	

CT440	411	112	2	[32-53]**7**[60-85]	

CT442	515	150	2	[38-61]**6**[67-88]	15 kDa Cysteine-Rich Protein (CrpA)

CT449	976	110	2	[42-63]**6**[69-87]	

CT483	316	121	2	[39-62]**20**[82-105]	

CT484	467	332	2	[29-51]**6**[57-80]	Tetratrico Peptide Repeats (TPRs)

CT556	496	159	2	[100-123]**12**[135-152]	

CT565	340	147	2	[49-81]**25**[106-135]	

CT616	286	429	2	[39-59]**6**[65-84]	UPF0242 extended coiled coils

CT618	612	266	2	[213-236]**6**[242-262]	

CT642	344	271	2	[167-188]**2[**190-205]	

CT788	597	166	2	[6-26]**7**[33-54]	

CT789	No ortholog	83	2	[12-30]**28[**58-78]	

CT813	978	264	2	[41-61]**7[**68-94]	coiled coils

CT814.1	160	120	3	[6-32]**20**[52-69]**28**[97-116]	

CT837	176	658	2	[543-561]**12**[573-592]	

CT850	151	405	4	[24-46]**2**[48-68] & [71-91]**4**[95-119]	extended coiled coils

CT873	No ortholog	105	2	[29-43]**2**[45-59]	

TOTAL	**59 proteins**				

(1) Number of the group of orthologs to which the protein belongs (2) total number of transmembrane segments detected by Polyphobius predictor algorithm (transmembrane segments identified as signal peptides are noted SP). (3) [first-last] amino acid of the transmembrane segment for each bilobal domain, the number of amino acids in the loop appears in bold. Additional features contain information extracted from genome annotations and from the present analysis.

**Table 3:** *C. pneumoniae *CWL029 putative Inc proteins, see Table 2 for details.

Sequence ID	0rtholog Group # (1)	Length (aa)	TM segments (2)	TM coordinates (3)	Additional feature(s)
CPn0007	688	964	**6**	[7-30]**10**[40-61] & [116-140]**10[**150-171] & [226-250]**6**[256-281]	DUF1978 coiled coils

CPn0010	688	297	**2**	[2-16]**9**[25-46]	extended coiled coils

CPn0011	688	241	**2**	[30-53]**6**[59-85]	extended coiled coils

CPn0026	no ortholog	288	**2**	[37-63]**5**[68-89]	

CPn0028	950	261	**3**	[48-64]**9**[73-87]**2**[89-103]	DUF648

CPn0034	908	416	**2**	[52-76]**6**[82-105]	Macro domain

CPn0041	688	449	**2**	[2-16]**9**[25-46]	DUF1978 coiled coils

CPn0043	688	642	**2**	[29-52]**7**[59-80]	DUF1978 extended coiled coils

CPn0045	688	574	**2**	[2-16]**9**[25-46]	DUF1978 coiled coils

CPn0049	505	160	**3**	[49-69] & [102-118]**2**[120-141]	

CPn0065	774	576	**4**	[33-55]**6**[61-88] & [242-263]**6**[269-291]	coiled coils

CPn0066	462	577	**5**	[358-379]**14**[393-413]**12**[425-445] & [484-507] & [542-566]	DUF687

CPn0067	no ortholog	367	**2**	[34-58]**6**[64-84]	

CPn0069	462	617	**5**	[398-419]**15**[434-455]**10**[466-488] & [526-547] & [585-608]	DUF687

CPn0072	772	335	**3**	[91-109]**6**[115-135] & [166-194]	

CPn0124	688	485	**2**	[2-18]**10**[28-49]	DUF1978 coiled coils

CPn0126	688	759	**2**	[2-17]**10**[27-48]	DUF1978 extended coiled coils

CPn0130	no ortholog	165	**2**	[29-51]**5**[56-77]	

CPn0131	no ortholog	344	**2**	[36-55]**6**[61-81]	

CPn0132	no ortholog	325	**2**	[29-53]**6**[59-84]	

CPn0146	287	161	**3**	[6-25]**12**[37-59]**6**[64-91]	

CPn0147	455	149	**2**	[43-64]**6[**70-93]	

CPn0150	553	1537	**4**	[79-99]**6**[105-124] & [841-861]**6**[867-886]	extended coiled coils

CPn0157	916	142	**2**	[60-74]**2**[76-90]	DUF648

CPn0164	no ortholog	167	**2**	[37-58]**6**[64-86]	

CPn0166	no ortholog	111	**2**	[35-58]**6[**64-86]	

CPn0169	no ortholog	264	**2**	[32-53]**6**[59-80]	

CPn0173	no ortholog	91	**2**	[37-55]**7**[62-79]	

CPn0174	568	156	**2**	[50-74]**11**[85-107]	coiled coils

CPn0181	no ortholog	133	**2**	[73-91]**16**[107-125]	

CPn0186	264	390	**2**	[38-62]**6**[68-88]	similarity to CT119 IncA extended coiled coils

CPn0203	916	265	**2**	[25-43]**20**[63-82]	DUF648

CPn0211	no ortholog	98	**2**	[37-61]**7**[68-91]	

CPn0212	no ortholog	393	**2**	[38-61]**6**[67-88]	

CPn0214	no ortholog	404	**2**	[2-20]**9**[29-52]	coiled coils

CPn0215	no ortholog	419	**2**	[31-57]**6**[63-88]	extended coiled coils

CPn0216	no ortholog	145	**2**	[25-51]**6**[57-81]	

CPn0221	804	136	**3**	[52-72]**12**[84-105]**5**[110-130]	

CPn0223	461	126	**2**	[54-72]**26**[98-123]	

CPn0225	no ortholog	223	**2**	[32-55]**9**[64-89]	coiled coils

CPn0226	804	134	**2**	[56-76]**14**[90-121]	

CPn0230	682	226	**2**	[41-65]**12**[77-100]	

CPn0240	713	388	**4**	[79-93]**12**[105-133] & [188-215]**7**[222-248]	

CPn0241	713	384	**4**	[88-104]**11**[115-144] & [201-228]**6**[234-261]	

CPn0242	63	144	**3**	[30-54] & [92-113]**10**[123-141]	

CPn0243	63	141	**3**	[29-53] & [94-115]**8**[123-13]	

CPn0266	129	231	**4**	[33-57]**11**[68-90] & [124-146]**6**[152-173]	

CPn0267	129	263	**4**	[37-62]**6**[69-94] & [156-178]**6**[184-205]	

CPn0277	no ortholog	169	**2**	[78-100]**9**[109-139]	

CPn0284	no ortholog	165	**2**	[33-56]**2**[58-82]	

CPn0285	475	515	**2**	[32-54]**11**[65-86]	

CPn0288	132	382	**6**	[61-82]**8**[90-113] & [182-203]**2**[205-225]**23**[248-262]**2**[264-278]	

CPn0291	79	176	**2**	[100-129]**7**[136-166]	Inclusion Membrane Protein B (IncB)

CPn0292	26	203	**2**	[127-151]**19**[170-193]	Inclusion Membrane Protein C (IncC)

CPn0308	no ortholog	121	**2**	[29-53]**6**[59-81]	

CPn0312	236	151	**3&SP**	[SP 1-24] [37-55] & [94-112]**6**[118-137]	

CPn0334	580	171	**3&SP**	[SP 1-26] [50-77]**17**[94-118]**19**[137-158]	

CPn0334	580	171	**3&SP**	[SP 1-26] [50-77]**17**[94-118]**19**[137-158]	

CPn0352	654	419	**2**	[32-51]**6**[57-80]	DUF1389

CPn0354	no ortholog	447	**2**	[26-50]**2**[52-74]	DUF1389

CPn0355	no ortholog	433	**2**	[28-48]**6**[54-77]	DUF1389

CPn0357	no ortholog	283	**2**	[27-52]**5**[57-76]	DUF1389

CPn0365	no ortholog	339	**2**	[33-54]**6**[60-79]	

CPn0366	no ortholog	155	**2**	[49-71]**14**[85-112]	

CPn0367	722	245	**2**	[40-61]**6**[67-86]	Macro domain

CPn0369	722	404	**2**	[39-60]**6**[66-86]	Macro domain

CPn0370	722	371	**2**	[36-56]**5**[61-80]	Macro domain

CPn0371	no ortholog	119	**2**	[43-65]**6**[71-92]	

CPn0372	no ortholog	105	**2**	[36-62]**6**[68-90]	

CPn0375	no ortholog	165	**4**	[49-73]**6**[79-103]**9**[112-131]**6**[137-155]	

CPn0381	462	591	**5**	[372-393]**15**[408-428]**11**[439-459] & [498-521] & [557-581]	DUF687

CPn0404	402	339	**3**	[123-137]**2**[139-153]**4**[157-174]	

CPn0431	901	111	**2**	[39-60]**11**[71-92]	

CPn0432	no ortholog	101	**2**	[36-59]**6**[65-86]	

CPn0440	431	212	**2**	[54-72]**6**[78-101]	coiled coils

CPn0442	485	172	**3**	[44-75]**12**[87-111]**6**[117-137]	

CPn0443	667	417	**4**	[53-74]**6**[80-102]**12**[114-138]**6**[144-165]	

CPn0458	no ortholog	695	**2**	[6-22]**23**[45-66]	DUF562 and DUF575

CPn0474	141	589	**10**	[58-81]**6**[87-109]**20**[129-150]**15**[165-183]**29**[212-237]**6**[243-266] & [345-367]**6**[373-400]**12**[472-492]**6**[498-518]	

CPn0480	238	218	**2**	[101-127]**10**[137-159]	

CPn0481	no ortholog	536	**2**	[31-52]**6**[58-84]	

CPn0517	no ortholog	279	**2**	[39-63]**7**[70-93]	

CPn0523	898	110	**2**	[26-47]**2**[49-73]	

CPn0524	800	359	**2**	[35-57]**6**[63-82]	Macro domain

CPn0537	160	119	3	[6-32]**21**[53-69]**27**[96-115]	

CPn0554	411	96	**2**	[33-55]**9**[64-87]	

CPn0556	515	196	**2**	[74-98]**6**[104-125]	15 kDa Cysteine-Rich Protein (CrpA)

CPn0565	106	366	**2**	[39-58]**6**[64-85]	

CPn0585	842	651	**2**	[52-75]**6**[81-103]	extended coiled coils

CPn0601	316	106	**2**	[36-61]**12**[73-101]	

CPn0602	467	334	**2**	[29-51]**6**[57-80]	Tetratrico Peptide Repeats (TPRs)

CPn0753	612	282	**2**	[228-250]**6**[256-276]	

CPn0755	286	401	**2**	[14-34]**6**[40-58]	UPF0242 extended coiled coils

CPn0770	344	264	**2**	[161-181]**2**[183-199]	

CPn0822	340	158	**3**	[46-60]**2**[62-83]**20**[103-134]	

CPn0829	no ortholog	185	**2**	[108-131]**18**[149-180]	

CPn0830	no ortholog	172	**3**	[6-22] & [91-115]**16**[131-163]	

CPn0834	496	154	**2**	[98-121]**12**[133-150]	

CPn0882	462	379	**5**	[169-188]**7**[195-217]**13**[230-247]**20**[267-285] & [334-358]	DUF687

CPn0930	no ortholog	158	**2**	[28-51]**5**[56-77]	

CPn0938	597	158	**2**	[2-22]**6**[28-50]	

CPn0994	176	681	**2**	[553-571]**12**[583-602]	coiled coils

CPn1008	151	432	**4**	[34-55]**2[**57-77]**3**[80-99]**6**[105-130]	extended coiled coils

CPn1027	no ortholog	527	**2**	[34-58]**6**[64-89]	coiled coils

CPn1029	226	279	**6**	[28-46]**14**[60-84]21[105-132]**17**[149-175]**21**[196-217]**20**[237-263]	

CPn1051	no ortholog	103	**2**	[36-62]**5**[67-91]	

CPn1054	688	811	**2**	[28-52]**11**[63-83]	DUF1978 extended coiled coils

CPn1055	688	276	**2**	[2-16]**5**[21-46]	DUF1978 extended coiled coils

TOTAL	**107 proteins**				

We next studied the evolutionary relationship of the putative Inc proteins using InParanoid/Multiparanoid programs, which can automatically find orthology relationships between proteins in multiple proteomes [34]. From the 537 Inc-candidates sequences, 126 are "orphan" sequences, showing no orthology relationship with other putative Inc. Most of these orphan putative Inc proteins are from *P. amoebophila *(68 sequences) and from *C. pneumoniae *(36 sequences). The remaining 411 putative Inc proteins come into 109 groups of orthologs. Interestingly, 50 and 21 of the ortholog groups were specific of the *Chlamydophila *and of the *Chlamydia *families, respectively (Figure 2B). This suggests that many Inc proteins might fulfill species-specific or family-specific functions. Alternatively, and not exclusively, Inc proteins that are involved in similar functions in distinct species might not be recognizable at the primary sequence level.

Genes coding for Inc proteins are scattered in the genomes with a few "hot spots" that cluster several consecutive *inc *genes (see in Figure 2C the distribution of *C. trachomatis inc *genes as an example). Transcription of the genes in operons has been demonstrated in a few cases [14,15]. Finally, Inc proteins have an average length of 279 residues (median: 207, ranging from 61 to 1537 residues). Most members of the family have only two transmembrane segments. Inc proteins with four transmembrane segments have been observed [16,26,35], but the existence of Inc proteins with more than four transmembrane segments remains to be confirmed experimentally.

### Experimental validation of the results

**Figure 3:**
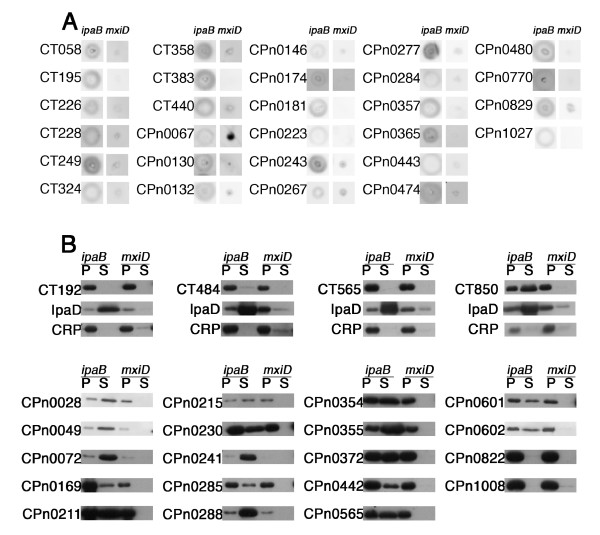
**Identification of type III secretion signals in putative Inc proteins**. A. Secretion assay on colonies. The *ipaB *(left) or *mxiD *(right) strains of *S. flexneri *were transformed with different *Chlamydia*/Cya constructs, isolated, and one colony for each construct was grown overnight in contact with a PVDF membrane, which served the following day to reveal the localization of the reporter protein using anti-Cya antibodies. All chimera shown in this figure carry a functional TTS assay, which allow the chimera to diffuse in a halo in the *ipaB *strain but not in the *mxiD *strain. B. Secretion assay in liquide cultures. Exponential cultures of *ipaB *or *mxiD *strains expressing the indicated chimeras were fractionated. The supernatant (S) and pellet (P) fractions were run on SDS-PAGE and western blot was performed using anti-Cya antibody. Membranes were later probed again using anti IpaD and anti CRP antibodies, to check that there was no bacterial lysis and that TTS was functional in the *ipaB *strain. These controls were systematically performed and are only shown for the first row of constructs tested. The supernatant fractions is concentrated 25-fold compared to the pellet fraction. Note that CPn0169/Cya is detectable in the culture supernatant, but in very low proportion relative to its very high expression level. This is unlike other secreted chimera, we therefore concluded that this protein does not carry a functional TTS signal.

We had previously shown that three *C. pneumoniae *and nine *C. trachomatis *Inc proteins had an amino-terminal sequence that was recognized as a TTS signal in *Shigella flexneri*, strongly suggesting that TTS is the mechanism by which Inc proteins are exported to the inclusion surface [24,36]. This property, which is independent of the characteristics of Inc proteins on which the biocomputing approach was based, was used to validate our *in silico *results. We included in the experiment 16 of the *C. trachomatis *and *C. pneumoniae *putative Inc proteins for which we had localization data and which had not been previously tested in the *Shigella *assay (Table 1 and 4). Because such data are scarce in the case of *C. pneumoniae*, we also included putative Inc proteins. Those were randomly chosen except for CPn0284 and CPn0285, which were included because they had not been observed on the inclusion membrane [37]. To determine whether putative Inc proteins contained a TTS signal we constructed chimeras between the amino-terminal part of the putative Inc proteins and a reporter protein, the calmodulin-dependent adenylate cyclase (Cya). Constructs were introduced into *S. flexneri *strains expressing various phenotypes with respect to type III secretion, i.e. in which secretion was constitutively turned on (*ipaB *mutant) or deficient (*mxiD *mutant). Secretion was assayed on colonies grown on agar plates: secreted chimera diffuse in the agar during overnight growth of the colony, while non-secreted chimera remain associated to the bacteria. After transfer on a nitrocellulose membrane and western blot against the Cya reporter protein, the secreted chimera appear as a halo around the colony, while the non secreted constructs are only visible at the spot where the colony grew [36]. About half of the chimeras were seen to be translocated by a TTS dependent process by this assay (Figure 3A). All chimeras that did not show a secretion pattern in the colony assay were tested again in liquid culture conditions [24], which is slightly more sensitive, to exclude the possibility that secretion occurred but was below detection level with the secretion assay on colonies. After subcellular fractionation of a culture of the *ipaB *or *mxiD *strains transformed with a chimeric construct, the presence of the chimera was assayed by western blot in the pellet and supernatant fractions. Seventeen out of the 23 chimera tested in this assay were found in the supernatant when expressed in the *ipaB *strain and not in the *mxiD *strain (Figure 3B). To verify that the presence of the chimera in the supernatant was not due to bacterial lysis, the fractions were also probed with an antibody against the cytosolic cyclic AMP receptor protein (CRP). Finally, probing the membranes with an antibody against the endogenous type III secretion substrate IpaD showed that type III secretion was functional in each of the transformed *ipaB *cultures. Therefore, absence of the chimera in the supernatant of these cultures did not result from a general defect in secretion but from the absence of a functional type III secretion signal in the chimera.

**Table 4:** Localization and presence of a Type III secretion signal in *C. trachomatis *and *C. pneumoniae *putative Inc proteins.

Sequence ID (*C. trachomatis*)	Localization	TTSS
CT005		

CT006		

CT018		

CT036		

CT058	Bacteria	**Y **(Figure 3A)

CT079		

CT081		

CT101	Inclusion	

CT115_IncD	Inclusion	**Y **[36]

CT116_IncE	Inclusion	**Y **[36]

CT117_IncF	Inclusion	

CT118_IncG	Inclusion	**Y **[36]

CT119_IncA	Inclusion	**Y **[36]

CT134		

CT135		

CT147	Inclusion	

CT164		

CT179		

CT192	Bacteria	**N **(Figure 3B)

CT195	Bacteria	**Y **(Figure 3A)

CT196		

CT214		

CT222	Inclusion	

CT223	Inclusion	**Y **[36]

CT224		

CT225	Inclusion	

CT226	Inclusion	**Y **(Figure 3A)

CT227		

CT228	Inclusion	**Y **(Figure 3A)

CT229	Inclusion	**Y **[36]

CT232_IncB	Inclusion	

CT233_IncC	Inclusion	**Y **[36]

CT249	Inclusion	**Y **(Figure 3A)

CT288	Inclusion	**Y **[36]

CT300		

CT324		**Y **(Figure 3A)

CT326		

CT345		

CT357		

CT358	Inclusion	**Y **(Figure 3A)

CT365		

CT383	Bacteria	**Y **(Figure 3A)

CT440	Inclusion	**Y **(Figure 3A)

CT442	Inclusion	**Y **[36]

CT449		

CT483		

CT484	Bacteria	**N **(Figure 3B)

CT556		

CT565	Bacteria	**N **(Figure 3B)

CT578		

CT616		

CT618	Inclusion	

CT642		

CT645		

CT788		

CT789		

CT813	Inclusion	

CT814.1		

CT837		

CT850	Inclusion	**Y **(Figure 3B)

CT873		

		

**Sequence ID** **(*C. pneumoniae*)**	Localization	TTSS

CPn0007		

CPn0010		

CPn0011		

CPn0026		**Y **[24]

CPn0028		**Y **(Figure 3B)

CPn0034		

CPn0041		

CPn0043		

CPn0045		

CPn0049		**Y **(Figure 3B)

CPn0065		

CPn0066		

CPn0067		**Y **(Figure 3A)

CPn0069		

CPn0072		**Y **(Figure 3B)

CPn0124		

CPn0126		

CPn0130		**Y **(Figure 3A)

CPn0131		

CPn0132		**Y **(Figure 3A)

CPn0146	Inclusion	**Y **(Figure 3A)

CPn0147	Inclusion	

CPn0150		

CPn0157		

CPn0164		

CPn0166		

CPn0169	**Bacteria**	**N **(Figure 3B)

CPn0173		

CPn0174		**Y **(Figure 3A)

CPn0181		**Y **(Figure 3A)

CPn0186	Inclusion	**Y **[24]

CPn0203		

CPn0211	**Bacteria**	**Y **(Figure 3B)

CPn0212		

CPn0214		

CPn0215		**Y **(Figure 3B)

CPn0216		

CPn0221		

CPn0223		**Y **(Figure 3A)

CPn0225		

CPn0226		

CPn0230	**Bacteria**	**Y **(Figure 3B)

CPn0240		

CPn0241		**Y **(Figure 3B)

CPn0242		

CPn0243		**Y **(Figure 3A)

CPn0266		

CPn0267		**Y **(Figure 3A)

CPn0277		**Y **(Figure 3A)

CPn0284	Bacteria	**Y **(Figure 3A)

CPn0285	Bacteria	**Y **(Figure 3B)

CPn0288		**Y **(Figure 3B)

CPn0291		**Y **[24]

CPn0292		**Y **[24]

CPn0308	Inclusion	**Y **[24]

CPn0312		

CPn0334		

CPn0352		

CPn0354		**Y **(Figure 3B)

CPn0355	**Bacteria**	**Y **(Figure 3B)

CPn0357	**Bacteria**	**Y **(Figure 3A)

CPn0365		**Y **(Figure 3A)

CPn0366		

CPn0367		

CPn0369		

CPn0370		

CPn0371		

CPn0372		**Y **(Figure 3B)

CPn0375		

CPn0381		

CPn0431		

CPn0432		

CPn0434		

CPn0440		

CPn0442		**Y **(Figure 3B)

CPn0443		**Y **(Figure 3A)

CPn0458		

CPn0474		**Y **(Figure 3A)

CPn0480		**Y **(Figure 3A)

CPn0481		

CPn0517	Inclusion	

CPn0523		

CPn0524		

CPn0537		

CPn0554		

CPn0556		

CPn0565		**Y **(Figure 3B)

CPn0585	Inclusion	**Y **[24]

CPn0601		**Y **(Figure 3B)

CPn0602	**Bacteria**	**Y **(Figure 3B)

CPn0753		

CPn0755		

CPn0767		

CPn0770		**Y **(Figure 3A)

CPn0822		**N **(Figure 3B)

CPn0829		**Y **(Figure 3A)

CPn0830		

CPn0834		

CPn0882		

CPn0930		

CPn0938		

CPn0994		

CPn1003		

CPn1008	**Bacteria**	**N **(Figure 3B)

CPn1027	Inclusion	**Y **(Figure 3A)

CPn1029		

CPn1051		

CPn1054		

CPn1055		

Novel localization data from this study are in bold. TTS data are from this study (Figure 3) or from previous study [36] and [24]. References for the localization data are found in Table 1 or in [18].

Table 4 combines these results and previous work [24,36]. Out of the 22 *C. trachomatis *putative Inc proteins that we tested, 19 (86%) possessed a functional TTS signal in their amino-terminal extremity. In *C. pneumoniae*, 44 putative Inc proteins were tested and the amino-terminal sequence of 41 (93%) were recognized as TTS signals in *S. flexneri*. Since the *C. pneumoniae *candidates were chosen randomly, this number can be extrapolated to the whole set of *C. pneumoniae *putative Inc proteins. It is very close to the proportion of TTS found in *C. trachomatis *putative Inc proteins, suggesting that the extrapolation is valid for all *Chlamydiaceae*, and that, overall, 90% of the putative Inc proteins that we identified based on their hydrophobic profile also possess a TTS signal.

### Identification of an ADP-ribose binding domain in several putative Inc proteins

Since Inc proteins are exposed to the host cytoplasm, we reasoned that they might present eukaryotic-like features. We used sensitive sequence analysis tools to search for conserved domains in putative Inc proteins, and in particular domains more abundant in eukaryotes. Proteins containing such a domain would not have been filtered out during the bioinformatics procedure if the domain covered only a restricted portion of the whole protein. Mimicry between Inc proteins and eukaryotic domains were reported in the case of CT147, whose overall structure resembles the early endosomal antigen-1 [26], CPn0585, which shows some similarity with Rab GTPase-interacting proteins [20], and IncA, which mimics SNARE domains [13]. These features were only noticed after careful sequence examination and cannot be revealed with standard sequence comparison tools. It is likely that primary sequence comparisons will fail to reveal the function of most Inc proteins, thus other methods need to be developed.

From their conservation within the *Chlamydiale *phylum 7 domains of unknown functions were identified in Inc proteins: DUF562 (in association with DUF575), DUF648, DUF687, DUF1978, DUF1389 and UPF0242. However, since these domains are only found in *Chlamydiales*, their identification does not give any clue on their putative function.

Interestingly, the only conserved domains we found were *macro *domains, which we discovered in 20 putative Inc proteins. The *macro *(or A1pp) domain is a module of about 180 amino acids which binds ADP-ribose and ADP-ribosylated proteins [38,39] and possibly a variety of related metabolites [40]. *Macro *domain proteins are found in eukaryotes, in bacteria, in archaea and in ssRNA viruses. While absent from the list of *P. amoebophila *putative Inc proteins, at least one *macro *domain was found in the six lists of *Chlamydiaceae *putative Inc proteins, and the motif appears to have expanded in the *Chlamydophila *lineage (Table 2, 3 and Additional files 1, 2, 3, 4, 5). The presence of a *macro *domain at the inclusion membrane could allow the bacteria to recruit NAD^+^-derived metabolites or ADP-ribosylated proteins to the inclusion membrane to fulfill various functions, depending on the specificity of these bacterial *macro *domains. However, the presence of a bacterial encoded *macro *domain at the inclusion membrane during infection remains to be confirmed by immunolocalization data, because the only member that has been investigated so far, CT058, was not detected at the inclusion [18].

### Secondary structure analysis of putative Inc proteins

We next analyzed the predicted secondary structure of putative Inc proteins. Excluding the bilobal hydrophobic domain from the calculation, 153 sequences out of 537 exhibited an alpha-helix content superior to 50%. Alpha helix-rich regions often constitute supersecondary structures such as coiled-coils and helical bundles and are encountered in many virulence effectors [41,42]. A very common structure mediating protein-protein interactions is the 34 amino acid helix-turn-helix motif formed by tetratricopeptide motif repeats (TPR) [43]. Using two prediction programs (Coils and Marcoils), we detected a number of alpha helix-rich Inc proteins with a high propensity to have coiled coil regions. Among those, 64 proteins in 9 ortholog groups are predicted to form extended (> 75 residues) coiled coil domains (Table 2, 3 and Additional files 1, 2, 3, 4, 5). The number of residues predicted to form coiled coils with a threshold of 50% (Marcoils) was found to be significantly enriched in the putative *C. trachomatis *Inc protein population compared to non Inc proteins with at least one transmembrane segment (Student's *t*-test t = 3,1, p < 0.0001)

The two programs sometimes generated different predictions, suggesting that the alpha helical structures may present discontinuities in the heptad pattern or organize into amphiphilic helix or solenoid superhelical structures. Indeed, most alpha-helices of more than 25 residues not predicted to form coiled coils adopt an amphiphilic conformation. In addition, seven sequences, all belonging to the same chlamydial specific ortholog group, are predicted to form solenoid superhelical structures characteristics of TPR repeats.

### Many *C. pneumoniae *putative Inc proteins are not translocated to the inclusion in the laboratory conditions

**Figure 4:**
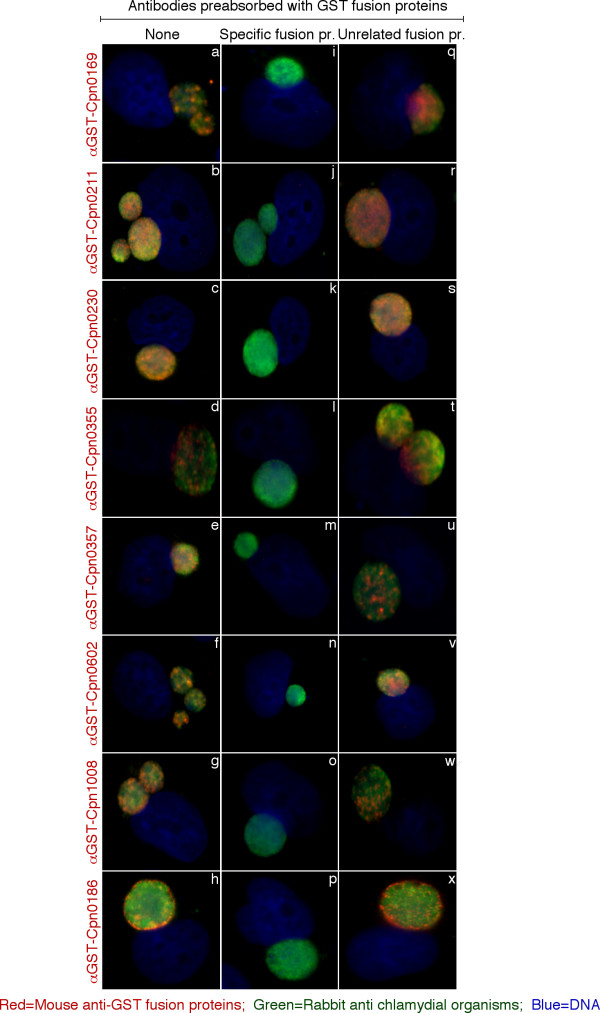
**Localization of 7 putative inclusion membrane proteins in *C. pneumoniae*-infected cells**. HeLa cells infected with *C. pneumoniae *AR39 for 96 hrs were immunostained with mouse anti-GST fusion protein antibodies plus a Cy3-conjugated goat anti-mouse IgG (red) and a rabbit anti-chlamydial organism antibody plus a Cy2-conjugated goat anti-rabbit IgG (green) and Hoechst to visualize DNA (blue). Antibodies against the GST-putative Inc fusion proteins detected signals inside the inclusions, overlapping with the chlamydial organisms. In contrast, antibodies against GST-CPn0186, a control Inc protein, showed peripheral labelling of the inclusion membrane (bottom panels). All antibody labelings were removed by preabsorption of the antibodies with the corresponding GST fusion proteins (panels i to p), but not the unrelated GST-fusion protein control (q to x).

Inc proteins were initially defined as chlamydial proteins that localized to the inclusion membrane during infection [10,14]. Later, the presence of at least one bilobed hydrophobic domain was identified as a feature common to all Inc proteins [16], and it is widely accepted that these two characteristics define the members of the family. Did our systematic search for proteins with a bilobed hydrophobic domain identify proteins that all localize to the inclusion membrane? The early work by Bannantine et al suggested a negative answer to this question since, out of the six putative Inc proteins investigated using specific antibodies, one (CT484) was associated with the bacteria but not the inclusion membrane [16]. We recently extended this observation showing that 5 additional *C. trachomatis *putative Inc were only found inside the inclusion [18] (see Table 4). These results show that the presence of a bilobed hydrophobic domain does not guarantee translocation to the inclusion membrane, at least for *C. trachomatis *Inc proteins. To know whether this result also applied to *C. pneumoniae*, we raised antibodies against 7 putative Inc proteins from *C. pneumoniae *(CPn0169, CPn0211, CPn0230, CPn0355, CPn0357, CPn0602 and CPn1008) as GST-tagged fusion proteins. As a control we used antibodies against the *C. pneumoniae *Inc protein CPn0186. The anti-fusion protein antibodies were used to localize the endogenous proteins in cells infected by *C. pneumoniae *for 96 hours. In contrast to the inclusion labeling observed with anti-CPn0186 antibodies, none of the 7 sera stained the inclusion membrane (Figure 4). The detection of endogenous antigens was removed by pre-absorption with corresponding GST fusion proteins but not heterologous GST fusion proteins, demonstrating the specificity of the antibodies. While they did not stain the inclusion membrane, the 7 sera labeled the bacteria, demonstrating that the corresponding proteins are expressed at this stage of infection, and remain bacteria-associated. We cannot exclude the possibility that some or all of these proteins are partially exposed on the membrane and not detected by this approach. However, we can conclude that these 7 putative Inc proteins are not constitutively secreted. Table 4 recapitulates the list of putative Inc proteins for these two species with the TTS and localization data.

## Discussion and Conclusions

Initially, to identify all putative Inc proteins, we started from the IncA domain from Pfam database (PF04156), which is derived from the multiple alignment of IncA-like sequences. This domain includes the hydrophobic domain and an adjacent coiled coil region, which are characteristics of IncA. When used to detect Inc proteins, this model misclassified Inc proteins sequences which are devoid of coiled coil regions and appeared far down in noise rank, with a non-significant Score/Evalue (e.g. IncB-C-D-E-F-G). This indicates that the Pfam IncA domain is too specific for a large scale genomic analysis. Known Inc proteins contain two transmembrane alpha-helical segments separated by a loop of less than 30 amino acids. Using this criteria and bioinformatics tools, we have searched for all putative Inc proteins in seven chlamydial proteomes and obtained 537 candidates. These results were validated experimentally for *C. trachomatis *and *C. pneumoniae*, as we found that 90% of the putative Inc proteins of these species had a TTS signal, which is a property of Inc proteins independent of their hydrophobicity profile.

Secondary structure analysis revealed that Inc proteins are enriched in coiled-coil domains. In bacteria, coiled-coil containing proteins represent 5% of proteins, and the majority contain only one helix of around 28 residues [44]. Extended coiled-coil domains are rare [45] and are enriched in type III (and type IV) secretion proteins [42]. Motor, membrane tethering, and vesicle transport proteins are the dominant eukaryote-specific long coiled-coil proteins, suggesting that coiled-coil proteins have gained functions in the increasingly complex processes of subcellular infrastructure maintenance and trafficking control of the eukaryotic cell [46]. Therefore, the abundance of sequences with a high probability for coiled-coil conformation among the putative Inc proteins supports the hypothesis that these proteins are exposed on the cytosolic side of the inclusion membrane where they may participate in controlling the interaction between the inclusion and the cellular compartments of the host and/or to the motion of the inclusion in the cell, as we have previously shown in the case of IncA [13].

We have identified a TTS signal in 90% of the 66 putative Inc proteins of *C. trachomatis *and *C. pneumoniae *that we have tested. This result confirms the robustness of our secretion assay, for which we had previously demonstrated that the rate of false positive was below 5% [36]. Approximately 10% of the 66 putative Inc proteins tested did not have a functional TTS signal. None of the five putative Inc proteins for which the secretion assay gave a negative result and for which we have localization data was detected on the inclusion membrane, suggesting that they might correspond to real negatives.

Three different methods have recently been made available to predict TTS signals in the amino-terminal part of proteins ([47,48] and http://gecco.org.chemie.uni-frankfurt.de/T3SS_prediction/T3SS_prediction.html. We found that 64% (38/59) *C. trachomatis *putative Inc proteins were predicted to possess a TTS signal by at least one of the three softwares, and 45% (27/59) by at least two. Thus, although clearly successful at recognizing TTS signals, the current predicting tools have a higher rate of false negative than our experimental secretion assay. Conversely, 3 of the 6 proteins in which we did not find a functional TTS were predicted to have one by one program, again pointing to the successes and limits of *in silico *detection tools for TTS signals.

The amino-terminal sequence of only about 10% of the putative Inc proteins, for each species, was not recognized for TTS in *S. flexneri *(CT192, CT484, CT565, CPn0169, CPn0822 and CPn1008). Several explanations for these negative results can be proposed: (i) these putative Inc proteins might have lost their ability to be secreted, (ii) the sequence we considered as coding for the N-terminal segment might not correspond to the real N-terminal segment (for example from sequencing or annotation errors), (iii) in the chimera, the N-terminal segment might not be presented in a conformation compatible with its recognition by the *S. flexneri *TTS machinery, leading to a false negative result in our assay. Interestingly, both orthologous proteins CT565 and CPn0822 were not recognized as TTS substrates, suggesting the TTS signal may have been lost before speciation of the two lineages. In contrast, CPn0602 has a functional TTS signal while the orthologous protein CT484 has none, suggesting that the ability to be secreted was lost in the *C. trachomatis *lineage only. The reverse might apply to CT850, which is secreted, while the homologous protein CPn1008 is not. Finally, the amino terminal part of CT192 is missing from other *C. trachomatis *serovars, as well as from the *C. muridarium *homolog, while the rest of the protein is very conserved. This might reflect the absence of evolutionary pressure to keep the amino-terminal domain compatible with TTS in this protein, suggesting that CT192 is not a TTS substrate.

Finally, in agreement with earlier observations [16,18], we observed that not all putative Inc proteins are detected on the inclusion membrane using specific antibodies. Localization data are now available for 16 *C. pneumoniae *putative Inc proteins. Only 7 of them (44%) were detected on the inclusion membrane (Table 4). If this number can be extrapolated to the whole genome, only about 47 out of the 107 putative *C. pneumoniae *Inc proteins might be exposed at the inclusion surface in the culture model we use (HeLa cells), meaning that the expansion of putative Inc proteins coded by *C. pneumoniae *genome does not necessarily correlate with an increase in the number of bacterial proteins exposed at the inclusion surface in this species. In comparison only 6 out of 29 (20%) *C. trachomatis *Inc proteins for which localization data were obtained were not detected at the inclusion membrane. This suggests that in this species the pool of 'non-translocated Inc proteins' might be smaller than in *C. pneumoniae*. However, the *C. trachomatis *proteins analyzed were not randomly chosen thus making the comparison difficult.

We showed that 3 out of the 6 putative *C. trachomatis *Inc proteins that were only detected on the bacteria had a functional TTS signal (in *C. pneumoniae*, 7 out of 9 such proteins had a functional TTS signal). Therefore, although some of these 'non-translocated Inc proteins' might correspond to false positives of the biocomputing approach, other explanations are needed to account for the absence of detection at the inclusion membrane of many putative Inc proteins. Firstly, it could be that only a small proportion of these putative Inc proteins is translocated and could be undetected by our method. Alternatively, they might be secreted very early in the developmental cycle. At early time points, it is difficult to distinguish between the inclusion and bacterial membranes and a transient appearance at the inclusion surface would be difficult to detect. Both scenarios raise the question of the difference between 'poorly' or 'transiently' translocated Inc proteins and other Inc proteins. Alternatively, 'non-translocated Inc proteins' might correspond to former inclusion proteins that have lost their function as such and are no longer secreted. Considering the drastic genome reduction observed in all chlamydiae, the maintenance of these genes imply that all of these proteins must have acquired another intrabacterial function, which makes this explanation very unlikely. Another hypothesis is that translocation of some Inc proteins is controlled and responds to unknown stimuli, which are absent from the culture conditions used here. In other bacteria, many TTS substrates are stored, usually in complex with chaperone proteins, before translocation by the TTS apparatus upon stimulation [49]. In addition to their distribution in inclusion membranes, several Inc proteins were detected in purified bacteria, indicating that the Inc proteins might be stored to some extent before translocation [10,15]. We have shown that Inc proteins were not soluble when expressed in *E. coli*, suggesting that in chlamydiae unknown chaperone protein(s) might assist their folding and availability for translocation [24]. The observation that some putative Inc proteins are mostly found at the inclusion membrane while others are only detected in the bacteria suggest that different pools of Inc proteins exist, whose translocation into the inclusion membrane responds to different cellular environment, cell types or even hosts. Noticeably, the expansion of putative Inc proteins in the *C. pneumoniae *genome compared to *C. trachomatis *accounts for about one third of the difference in gene number between the two species. This may reflect the need for *C. pneumoniae *to adapt to more variable environments, consistent with the hypothesis that certain Inc proteins may only be exposed on the surface of the inclusion in a regulated manner.

## Methods

### Sequence analysis

#### Proteomes data set

The protein sequences were retrieved from completely sequenced genomes of the following chlamydial species: *C. abortus *S26 3, *C. muridarum *strain Nigg, *C. pneumoniae *CWL029, *C. trachomatis *serovar D/UW-3/CX, *C. caviae *GPIC, *C. felis *Fe/C-56, *Candidatus '*Protochlamydia amoebophila' UWE25. Chlamydial proteomes were retrieved from the Comprehensive Microbial Resource (CMR) site.

**Analysis of hydrophobic domains **was conducted for membrane protein secondary structure prediction by the SPLIT program [28] and for topology analysis with Topcons program, which combines results of several predictors to yield a more reliable result [31].

**Clustering of Orthologs: **groups of ortholog in the seven genomes/proteomes were obtained using the All-versus-All sequences comparison InParanoid method and its extention MultiParanoid, which merge multiple pairwise ortholog groups from InParanoid into multi-species ortholog groups [34,50]. Each group of orthologs was given a number, which is reported in Tables 2, 3 and Additional files 1, 2, 3, 4, 5.

**Transmembrane protein **were collected with the Polyphobius program which combines transmembrane detection and signal peptide prediction. The method makes an optimal choice between transmembrane segments and signal peptides, and also allows constrained and homology-enriched predictions [33]. To reduce misclassification, proteins with a single transmembrane domain and a signal peptide were analyzed manually.

**Protein domain detection **were performed with rpsblast program (Blast package v2.2.19) [51] using the NCBI Conserved Domain Database (CDD, v2.18) [52]). Specific searches of domains were performed with the Hmmer package [53,54].

#### Multiple alignment and domain detection

Multiple sequence alignments were performed with the PralineTM program, which optimizes the information for each of the input sequences (predicted secondary structure and transmembrane structure) [55].

**Charge distributional analysis **was performed with SAPS [56].

### Secondary structure analysis

Secondary structure prediction was performed with the Proteus program (v2) [57]. To optimize the selection of proteins with coiled-coil regions we used two different approaches: firstly the Coils program [58] with windows of 28, 21 and 17 residues, and secondly the Maircoil program [59]. We considered high coiled coil predictions when both algorithms returned high probabilities of coiled coils. Other alpha helical conformations were predicted respectively with Heliquest for amphiphilic conformations [60] and TPRpred [61] for superhelical topologies as Tetratrico Peptide Repeats, Pentratrico repeats and SEL1-like repeats. Presence of Leucine zippers in coiled coils proteins were performed using 2ZIP [62].

### Type III secretion assays

Genomic DNA from *C. pneumoniae *strain TW183, *C. trachomatis *serovar D/UW-3/CX and *C. caviae *strain GPIC was prepared from bacteria extracted from infected HeLa cells, using the RapidPrep Micro Genomic DNA isolation kit (Amersham Pharmacia Biotech). Chimera comprising the 5' part of different chlamydial genes upstream of the gene coding for the adenylate cyclase of *Bordetella pertussis *were constructed by PCR as described [24]. The constructs include about 30 nucleotides upstream from the proposed translation start sites and the first 20 to 40 codons of the chlamydial genes. The chimeric constructs were transformed in the *S. flexneri *strains SF401 and SF620, which are derivatives of M90T in which the *mxiD *and *ipaB *genes, respectively, have been inactivated [63,64]. Secretion on liquid cultures [24] and on colonies [36] were assayed as described previously. Antibodies against the cyclic AMP receptor protein (CRP) and against IpaD were kindly given by A. Ullmann and C. Parsot, respectively (Institut Pasteur).

### Chlamydial gene cloning, fusion protein expression and antibody production

The ORFs encoding putative inclusion membrane proteins from the *C. pneumoniae *AR39 genome http://stdgen.lanl.gov/ were cloned into the pGEX vectors (Amersham Pharmacia Biotech) and expressed as fusion proteins with a glutathione-S-transferase (GST) fused at the N-terminus of the chlamydial proteins as previously described [65,66]. The expression of these proteins was induced by the addition of IPTG (Invitrogen) and the fusion proteins were extracted by lysing the bacteria via sonication in Triton X-100 lysis buffer (1% Triton X-100, 1 mM PMSF, 75 units ml^-1 ^aprotinin, 20 mM leupeptin and 1.6 mM pepstatin). The GST fusion proteins were purified using glutathione-conjugated agarose beads (Pharmacia). The purified fusion proteins were used to immunize mice for producing polyclonal antisera [67]. The sera were collected and stored at -20°C until use.

#### Immunofluorescence assay

Monolayers of HeLa 229 cells grown on glass coverslips were infected with *Chlamydia pneumoniae *AR39 at a m.o.i. of 0.5 in the presence of 2 μg/ml cycloheximide. The chlamydial organisms and infection procedures were as described elsewhere [65,68]. Ninety-six hours after infection cells were fixed with 2% paraformaldehyde (Sigma) dissolved in PBS for 30 min at room temperature, followed by permeabilization with 0.1% Triton in PBS for an additional 10 min. After washing and blocking, the cell samples were subjected to antibody and chemical staining. Hoechst (blue; Sigma) was used to visualize DNA. A rabbit anti-chlamydial organism antibody (R12AR39, raised with *C. pneumoniae *AR39 organisms; unpublished data) plus a goat anti-rabbit IgG secondary antibody conjugated with Cy2 (green; Jackson Immuno-Research Laboratories) was used to visualize chlamydial inclusions. The polyclonal mouse antibodies raised against putative inclusion membrane *C. pneumoniae *GST fusion proteins plus a goat anti-mouse IgG conjugated with Cy3 (red; Jackson ImmunoResearch) were used to visualize the corresponding antigens. In some cases, the primary antibodies were pre-absorbed with either the corresponding or heterologous fusion proteins immobilized onto agarose beads (Pharmacia) prior to staining cell samples. The preabsorption approach was carried out by incubating the antibodies with bead-immobilized antigens overnight at 4°C followed by pelleting the beads. The remaining supernatants were used for immunostaining. The immunofluorescence images were acquired with an Olympus AX-70 fluorescence microscope equipped with multiple filter sets (Olympus) as described previously [69-71]. Briefly, the multi-colour-labelled samples were exposed under a given filter set at a time and single color images were acquired using a Hamamatsu digital camera. The single color images were then superimposed with the software SimplePCI. All images were processed using Adobe Photoshop (Adobe Systems).

## Authors' contributions

PD performed sequence analyses and wrote the manuscript, RF performed the localization study and wrote the manuscript, AS conceived the study, carried out secretion assays and wrote the manuscript, CD and GZ participated in the design of the study. All authors read and approved the final manuscript.

## Supplementary Material

Additional file 1 **Table S1: Putative Inc proteins from *C. muridarium***. Click here for file

Additional file 2 **Table S2: Putative Inc proteins from *C. felis***. Click here for file

Additional file 3 **Table S3: Putative Inc proteins from *C. caviae***. Click here for file

Additional file 4 **Table S4: Putative Inc proteins from *C. abortus***. Click here for file

Additional file 5 **Table S5: Putative Inc proteins from *Candidatus '*Protochlamydia amoebophila' UWE25**. Click here for file
